# The “Child Health Evidence Week” and GRADE grid may aid transparency in the deliberative process of guideline development

**DOI:** 10.1016/j.jclinepi.2012.03.004

**Published:** 2012-09

**Authors:** Newton Opiyo, Sasha Shepperd, Nyokabi Musila, Mike English, Atle Fretheim

**Affiliations:** a KEMRI-Wellcome Trust Research Programme, P.O. Box 43640, Nairobi 00100, Kenya; b Section for International Health, Institute of Health and Society, University of Oslo, P.O. Box 1130, Blindern 0318, Oslo, Norway; c Department of Public Health, University of Oxford, Headington, OX37LF, Oxford, UK; d Department of Pediatrics, University of Oxford, John Radcliffe Hospital, Headington, Oxford, OX3 9DU, UK; e Global Health Unit, Norwegian Knowledge Centre for Health Services, P.O. Box 7004 St. Olavs plass, N-0130, Oslo, Norway

**Keywords:** Clinical practice guidelines, Evidence, Knowledge translation, Transparency, GRADE, Pediatrics

## Abstract

**Objective:**

To explore the evidence translation process during a 1-week national guideline development workshop (“Child Health Evidence Week”) in Kenya.

**Study Design and Setting:**

Nonparticipant observational study of the discussions of a multidisciplinary guideline development panel in Kenya. Discussions were aided by GRADE (Grading of Recommendations Assessment, Development, and Evaluation) grid.

**Results:**

Three key thematic categories emerged: 1) “referral to other evidence to support or refute the proposed recommendations;” 2) “assessment of the presented research evidence;” and 3) “assessment of the local applicability of evidence.” The types of evidence cited included research evidence and anecdotal evidence based on clinician experiences. Assessment of the research evidence revealed important challenges in the translation of evidence into recommendations, including absence of evidence, low quality or inconclusive evidence, inadequate reporting of key features of the management under consideration, and differences in panelists’ interpretation of the research literature. A broad range of factors with potential to affect local applicability of evidence were discussed.

**Conclusion:**

The process of the “Child Health Evidence Week” combined with the GRADE grid may aid transparency in the deliberative process of guideline development, and provide a mechanism for comprehensive assessment, documentation, and reporting of multiple factors that influence the quality and applicability of guideline recommendations.

## 1 Background

**What is new?**

• A common limitation in guideline development is the lack of transparency when translating evidence into recommendations.
• The findings give insights into the many factors that influence the decision-making process in multidisciplinary guideline panels.
• GRADE (Grading of Recommendations Assessment, Development, and Evaluation) grid may aid transparency and provide a mechanism for linking multiple factors that influence the quality and applicability of guideline recommendations.
• The “Child Health Evidence Week” may provide an efficient and inclusive rapid guideline development model in low-income countries.

There is a broad agreement that clinical practice guidelines should be “evidence based” but there has been less agreement on how to achieve this. In response to criticism that their process of guideline development has not always been made explicit [1], the World Health Organization (WHO) recently indicated that wherever possible its guidance should be supported by rigorous reviews of the evidence that has been critically appraised using the GRADE (Grading of Recommendations Assessment, Development, and Evaluation) tool [2]. This approach (and others) [3–6] recognize that the evidence must be viewed in the context of any relevant local evidence (e.g., microbial resistance patterns), what is feasible in the local clinical setting and what is acceptable to intended users (health care professionals) and patients.

Integrating global research evidence with locally relevant evidence and contextual factors has, however, rarely been undertaken in an explicit or structured fashion [1,7–9]. This is a particular problem in low-income countries (LICs). For newborn and child health, current guidance in LICs is mainly derived from that provided by WHO and its global partners (e.g., The United Nations Children’s Fund, UNICEF). The process of incorporating such guidance into national guidelines, often referred to as “adaptation,” is rarely described, and the roles that value-based judgments and context-specific information play in developing recommendations are often not clear.

### 1.1 Aim

We took advantage of efforts in Kenya to revise national guidelines for newborn and pediatric hospital care to examine the process of creating recommendations guided by the GRADE approach. The aim of the study was to document and characterize the discussions that took place among a multidisciplinary group of health care professionals who attended a week long meeting to review evidence and make guideline recommendations. Specifically, we explored the following two topics.

• Which aspects of the quality and nature of research evidence were key in shaping discussions?
• Which aspects of local contextual factors influenced the acceptance or rejection of evidence and the final recommendations?

## 2 Methods

### 2.1 Design

This was a descriptive study of the development of national guidelines for the management of common newborn and childhood illnesses in Kenya. Discussions between stakeholders attending a 5-day national guideline development workshop (“Child Health Evidence Week”) held between 21st and 25th, June 2010, were observed, recorded, analyzed, and interpreted.

### 2.2 Participants

The organizers of the guideline development workshop (that included N.O. and M.E.) used a purposive sampling approach to select the guideline development panel after consultations with relevant stakeholders involved in the provision of newborn and pediatric care services in Kenya. Briefly, the Kenya Medical Research Institute (KEMRI)-Wellcome Trust Research Program, working with the Ministry of Medical Services (MoMSs) identified leading individuals or key institutions of relevance to inpatient newborn and pediatric care (health worker training schools; professional associations; international agencies; national research institutes; and national-, regional-, and district-level facilities). The MoMSs then sent letters requesting these individuals or those nominated by their departmental heads to join the guideline development panel. The invitation letters described the expected roles of the participants (e.g., prereading of provided evidence summaries, time commitments) and the research component of the workshop. Aiming at wide representation, the guidelines development panel finally consisted of 70 individuals (Table 1).

### 2.3 Child Health Evidence Week

The “Child Health Evidence Week” was a 1-week meeting of neonatal and pediatric stakeholders gathered to develop evidence-based guidelines for the hospital management of common newborn and childhood illnesses in Kenya [10]. The clinical questions addressed were identified after consultations between KEMRI-Wellcome Trust Research Program, Kenyan MoMSs, technical experts (e.g., local WHO representatives), and other relevant stakeholders (e.g., practitioners and professional societies).

The identification process was informed by existing national guidelines (as outlined in the Basic Pediatric Protocols [11]), and focused on clinical areas where there was 1) lack of clear policy (e.g., hand washing); 2) controversy on acceptable best practice (e.g., newborn feeding regimens); or 3) likely need to revise or change existing recommendations given recent research findings (e.g., malaria). After this process, 11 clinical topics addressing assessment, diagnosis, and treatment of priority newborn and childhood conditions were identified (Appendix C on the journal’s Web site at www.jclinepi.com). Evidence summaries and draft recommendations were prepared for each of the clinical topics by a core team of 14 researchers and clinicians working in groups of 2 or 3. Evidence was summarized and packaged in three formats: systematic reviews, systematic reviews with summary-of-findings tables, and “graded-entry” formats (a “front-end” summary of key information linked to a contextually framed short narrative report plus the full systematic review) (see Appendix D on the journal’s Web site at www.jclinepi.com, e.g., “front-end” summary [10]). The evidence summaries were sent to participants 1 month before the workshop.

Participants were introduced to the GRADE approach and the proposed procedures for the development of recommendations on the first day of the workshop. During the subsequent days, the core team members made presentations on the evidence underlying proposed recommendations, using the PICO (Patient, Intervention, Comparator, Outcome) format and GRADE method to summarize the quality of the evidence and introduce possible additional considerations that might impact on the strength of recommendations. Each presentation was followed by a facilitated discussion. This initially focused on the formal evidence presented and subsequently, after presenting a draft recommendation, the wider issue of locally appropriate recommendations. Draft recommendations were then amended where necessary, and participants invited to vote for or against proposed recommendations. The voting process was aided by a modified GRADE grid [12], a scaled polling table that allows participants to anonymously record their approval or disapproval of a proposed recommendation (Appendix A). Votes were counted and fed back to participants using power point to display bar graphs of the results allowing participants a final, short discussion before confirmation of a final recommendation. The presentation, discussion, revision of wording of recommendation, and voting took approximately 2 hours for each of the clinical topics addressed. The deliberative process was facilitated by one, nonvoting investigator (M.E.).

### 2.4 Data collection

Three investigators (A.F., S.S., and N.M.) independently observed and recorded comments made during the full workshop, focusing on discussions about research evidence, practitioner experiences and values, and context-specific issues that might influence acceptability and implementation of proposed recommendations.

### 2.5 Data analysis

Three investigators (A.F., S.S., and N.M.) independently reviewed their field notes of panel discussions, and grouped comments into a number of clusters. Groupings were guided by previously identified criteria for assessing the applicability of systematic review evidence [13]. Themes emerging from these initial, independent analyses were then compared and discussed iteratively among the investigators (A.F., S.S., N.M., and N.O.) until a first common set of descriptive themes was identified. For each main theme, we identified related subthemes that provided more depth and detail to our description of the content of the discussions. A table summarizing these initial themes and subthemes was then prepared by one of the investigators (N.O.); the other investigators (S.S., N.M., and A.F.) commented on this draft and a final set of themes (supported by extracts from the field notes) was arrived at. Further exploratory analysis was completed by tabulating the frequency of aspects of the identified themes for each of the clinical topics. Disagreements were discussed and resolved by consensus after a review of the field notes.

### 2.6 Ethics

Ethical approval for the conduct of this study was granted by the KEMRI scientific committee and National Ethics Review Committee in Kenya (SSC Protocol No 1770). The project was also presented to the Norwegian Regional Committee for Medical and Health Research Ethics, which did not find ethical clearance necessary for this type of study.

## 3 Results

Overall, our observations of the deliberative process showed broad participation with most of the participants actively contributing in the debates. Outcomes of the deliberative process are illustrated with the pneumonia recommendation, where the panel voted against the proposal to replace benzyl penicillin/ampicillin with oral amoxicillin for the treatment of severe pneumonia despite moderate quality evidence suggesting equivalence between the treatments and additional factors favoring amoxicillin (e.g., lower cost, more convenient twice daily dosing (see Appendices A and B)). This unexpected voting outcome was probably because of the indirectness of the evidence [14].

Three key themes (Table 2) emerged from field notes documenting participants’ discussions:

1. “Referral to other evidence to support or refute a proposed recommendation.”
2. “Assessment of the presented research evidence.” This category captures comments by the panelists reflecting a deeper assessment of the available research evidence.
3. “Assessment of the local applicability of the evidence.” This category captures comments about whether the available evidence is applicable in the local setting.

The themes and subthemes with quotes from the field notes as illustrative examples are presented below.

### 3.1 Referral to other evidence to support or refute a proposed recommendation

#### 3.1.1 Subtheme a) Research evidence

For nine of the 11 clinical topics, participants referred to other sources of research evidence to support or refute the draft recommendations (Table 2). This included the potential benefits and harms associated with treatment options, estimates of the magnitude of benefit associated with treatments, absence of relevant evidence, and inconclusive evidence about the effectiveness of treatment options:

“*Studies have shown that aminophylline is not better than salbutamol*” (Asthma)

“*To demonstrate superiority of amoxicillin over co-trimoxazole, what is the absolute effect?*” (Pneumonia)

“*We had a study at Kenyatta [National Referral Hospital]. We did 8-hour Kangaroo care per day. Comparing with conventional care, we found difference in growth of babies, length of stay was a little bit shorter*” (Kangaroo care—unpublished report)

The absence of relevant evidence and inconclusive state of evidence were frequently noted as reasons for participants’ inability to cast a vote based on sound evidence for or against proposed recommendations:

“*We cannot say, [it] has not been tested. We are saving lives in the short-term. It is hard to act on this inconclusive evidence*” (HIV-Pneumonia)

“*Should we even give a recommendation? Given the evidence, there is not enough to provide guidance. The earlier concerns may be valid, we do not know. So perhaps we should not change*” (Malnutrition)

“*I beg to differ. We cannot apologize to these two patients if they die and say, we did not have the evidence*” (Asthma)

#### 3.1.2 Subtheme b) Clinical experience

Comments reflecting clinician’s experiences with proposed treatment options were observed in six of the 11 clinical topics (Table 2), and included experiences with routine clinical impacts of aspects of care, practical difficulties associated with treatments, and patient’s acceptability of proposed treatments:

“*In private sector I have experienced that you discharge already at 1.5* *kg with Kangaroo care, and they seem to do well*” (Kangaroo care)

“*I am worried about taking out aminophylline. I had a patient last week in status asthmaticus who I am convinced survived only because of the aminophylline infusion we gave him*” (Asthma)

“*In practice we use ready-to-use therapeutic foods [RTUFs] because we do not have F100. And it seems to be well accepted. If there are problems with the child taking it we mix e.g. with porridge*” (Severe malnutrition—RTUFs)

Of note, accounts of clinician’s experiences were observed more frequently in clinical conditions where existing practices were highly varied (e.g., initiation of feeds in sick newborns) and where the current research evidence base was weak or inconclusive (e.g., RTUF therapy used early among inpatients for severe malnutrition).

### 3.2 Assessment of research evidence

#### 3.2.1 Subtheme a) Quality of body of evidence

A range of issues reflecting participants’ scrutiny of the credibility of the evidence was noted in five of the 11 clinical topics (Table 2), and included sample size (power) issues, adequacy of available evidence on patient-relevant outcomes (e.g., mortality data), study execution (e.g., reliability of findings given premature study termination), adequacy of participant follow-up period, appropriateness of the study population (e.g., limited generalizability from recruiting inpatient populations), and measurement and selection of outcomes (e.g., potential biases associated with lack of blinding):

“*The numbers were small in this study, giving us power problems. And the unblinding gives us a problem with interpretation*” (Neonatal feeding regimens)

“*The trial was stopped early. Should we try and find out the reasons for the high mortality?*” (Malnutrition)

“*Is there mortality data on severe pneumonia?*” (Pneumonia)

“*In the absence of good quality evidence are there other good reasons for adopting the policy?*” (Pneumonia)

“*Selection bias of only studying children in hospital*” (HIV-Pneumonia)

Opinions diverged more frequently in clinical conditions where the quality of evidence was low (e.g., fluid resuscitation in severe malnutrition).

#### 3.2.2 Subtheme b) Nature of interventions

Comments seeking clarification of the features, or criticism of, the interventions being discussed were observed in nine of the 11 clinical topics (Table 2). Aspects discussed included definitions of the interventions, intensity of interventions, descriptions of any cointerventions, techniques relating to how the interventions were delivered, and the content of interventions:

“*What is the maximum duration of Kangaroo care?*” (Kangaroo care)

*Clarification required on sequence—wash* *→* *dry* *→* *use alcohol hand rub* (Hand washing)

*Question asked if inotropic support or mechanical ventilation were used: answer “no”* (Malnutrition)

“*Is ciprofloxacin recommended as p.o. [orally] or i.v. [intravenously]*” (HIV-Pneumonia)

“*For those on breast milk, were they on plain or with fortifier*” (Neonatal feeding regimens)

“*The various fluids have different components, and we have no research to guide as to which is the better*” (Malnutrition)

#### 3.2.3 Subtheme c) Interpretation of evidence

Comments alluding to differences in participants’ interpretation of evidence were observed in all clinical topics (Table 2), and included various opinions regarding: subgroup of populations to which results apply, range of factors explaining differences in study results, and outcome definitions. Additional comments highlighted challenges in the interpretation of study findings, for example, inconsistency of individual and pooled study results:

“*In these studies you are lumping all malnourished shock patients together, and this is a problem. There are likely to be important subgroups*” (Malnutrition)

“*Clarification on Papua New Guinea Study…showed no difference yet combination showed overall benefit of penicillin* *+* *gentamicin vs. chloramphenicol*” (Pneumonia)

“*What about the morbid condition of the child—was this considered?*” (Kangaroo care)

“*What was the definition of treatment failure?*” (Pneumonia)

“*The Duke study had a similar sample size to the Asghar study yet was unable to show a difference of effect*” (Pneumonia)

“*Given different patterns of resistance for different age groups, perhaps recommend cephalosporin for under 2-year olds, and penicillin plus chloramphenicol for the older ones?*” (Meningitis)

### 3.3 Assessment of the local applicability of evidence

#### 3.3.1 Subtheme a) Feasibility (implementation) issues

Reference to the likely barriers and facilitators to effective implementation (adoption) of the proposed treatment options were observed in eight of the 11 clinical topics (Table 2). Key factors discussed included costs of interventions, resource availability (including training), logistical issues, physical barriers, practical difficulties, and compliance issues. Also, observed were views related to adaptations required to facilitate effective delivery of proposed recommendations:

“*The science is there, fine. But if we change the recommendations we will need much training, for example, on the differentiation between the various severities of pneumonia*” (Pneumonia)

“*I think feasibility is major issue. A mother who has just delivered needs so many other things, and having a baby with oxygen, fluids…*” (Kangaroo care)

“*My concern is with the availability of IV [intravenous] salbutamol. If we take out aminophylline we have nothing*” (Asthma)

“*Getting vascular access will be difficult for people with limited training*” (Neonatal feeding regimens)

“*Compliance for amoxicillin could be a problem*” (Pneumonia)

“*…incubators are expensive and Kangaroo care will save money and space*” (Kangaroo care)

#### 3.3.2 Subtheme b) Knowledge of local clinical context

Comments where participants referred to knowledge about locally relevant practice-setting factors were observed in five of the 11 clinical topics (Table 2). This included awareness of local antimicrobial resistance patterns, local prevalence of febrile illnesses, and nature of available clinical skills:

“*The risk of development of resistance across 3rd generation cephalosporins is highly relevant also in our setting, as we have experienced at Kenyatta hospital where resistance has emerged*” (Neonatal sepsis—Antibiotics)

“*Many babies we receive at Kenyatta [National Referral Hospital] are hypothermic*” (Kangaroo care)

“*Nyanza—salmonella common in malaria endemic areas*” (Meningitis)

“*Outpatients in Kenya are staffed by clinical officers who are familiar with outpatient IMCI [Integrated Management of Childhood Illnesses]*” (Neonatal sepsis—Clinical signs)

#### 3.3.3 Subtheme c) Balance of benefit and harms

Comments reflecting judgments about likely benefits and harms of alternative treatments were observed in three of 11 clinical topics (Table 2), and included comparison of different types of benefits associated with treatments, likely benefits and harms, and considerations of benefits of treatments vs. resource consumption. Lack of cost data for most interventions seemed to limit explicit judgments about their net value:

“*Effective, safe, and accessible are the key components. If they are equally effective and safe, then accessibility is the issue*” (Asthma)

“*The issue here, as I understand it, is not effectiveness but safety and toxicity…and the issue is not cost, both are cheap*” (Asthma)

“*Costs of preparation of alcohol hand rub may be overweighed by costs of installing running water in all hospitals*” (Hand washing)

“*Should consider costs—direct costs of good hand hygiene as well as money spent on morbidity due to poor hand hygiene*” (Hand washing)

#### 3.3.4 Subtheme d) Clinician values, preferences, and acceptability

References to health worker perspectives, attitudes, cultural issues, preferences, and acceptability of proposed interventions were observed in four of the 11 clinical topics (Table 2):

“*My impression is that the attitude of the health professionals is an important factor*” (Asthma)

“*Personal preference of medicated soaps due to the perfume*” (Hand washing)

“*Need to consider cultural issues, e.g., uptake of alcohol hand rubs for Muslims*” (Hand washing)

“*Acceptability, F100 may not appear as sufficient to give to an older child as is just milk. RTUF is solid, so potentially more acceptable*” (Severe malnutrition—RTUFs)

## 4 Discussion

The realization that failure to use research findings in health care has a negative impact on patient’s care has led to an increased emphasis on identifying ways to transfer evidence into practice. The *Bellagio Child Survival Series* published in 2003 and the *Lancet Neonatal Survival Series* published in 2005 identified low-cost interventions with proven effectiveness for improving neonatal survival [15–17]. It is estimated that 35–66% of childhood and neonatal deaths in resource-poor settings could be prevented if there was high coverage of such interventions. One of the barriers to implementing these interventions is accessibility to and use of research evidence by policy makers and other intended users. In this study, we explored how research evidence and the related contextual issues inform the development of guideline recommendations in Kenya. A spectrum of factors were considered, these include the research evidence, implementation factors, and clinician values. The “Child Health Evidence Week” was a useful deliberative forum for disseminating key messages resulting from research syntheses, facilitating explicit discussions on research and context-specific data and values, and discussing draft recommendations during multidisciplinary guideline development.

The method for developing guidelines during the “Child Health Evidence Week” differed from the way guidelines are developed by established institutions in high-income countries [5,18]. Unlike these institutions, we had a larger panel (70 participants), a broader scope (11 clinical topics), and less time for deliberation (a 5-day meeting). Although such large panel sizes may limit individual participation rates, our observations revealed fairly balanced contributions owing probably to effective facilitation by an experienced, neutral chairperson. Further likely individual biases were minimized through the use of anonymous voting tool (GRADE grid) to reach consensus. We believe our relatively large panel was well suited to the broad focus, rapid nature of our task, and the need for inclusiveness in our guideline development process. Overall, our approach resulted in rapid production of evidence-based care recommendations on a broad package of interventions applicable in Kenya. With further refinement, this method may provide an efficient and inclusive guideline development model for use in other LICs.

Our observations revealed important challenges in the translation of evidence into recommendations. These included a lack of evidence, low quality or inconclusive evidence, limited cost data, inadequate reporting of features of treatments examined (e.g., durations and roles of supportive care), and differences in panelists’ interpretation of research literature. This last challenge can be an additional barrier to the timely achievement of consensus in multidisciplinary panels. Similar challenges have been documented in related studies [9,19,20]. Addressing these challenges may require a consensus-based process for deriving recommendations where relevant evidence is lacking or inconclusive [21,22], attention to details of aspects of care under consideration in evidence reviews (e.g., for a drug intervention, the dose, route, timing, and duration of administration needs to be reported) [23,24], and training of panel members in research synthesis relevant to guideline development.

Explicit discussions focusing on trade-offs between likely benefits, harms, and budget impacts of treatments were sparse. This confirms previous findings that the net value of treatments (despite being an important determinant of patient’s choices and preferences) is frequently overlooked by guideline development panels [25–27]. Similarly, despite the value of information about patient’s experiences with aspects of care (e.g., patients may attach different values to treatment outcomes) [28–30], explicit consideration of patient’s perspectives (or their surrogates such as parents and caretakers) was rarely observed. A possible reason for this was the absence of patient’s or consumer’s representatives in the convened panel (a shortcoming of our panel selection). Still, a number of practical difficulties remain concerning the feasibility and effectiveness of patient’s and consumer’s representation in guideline development panels [31–33]. Further studies are needed to define optimal presentation of patient-relevant information, and how best to incorporate patient’s perspectives in the guideline development process especially in low-income settings.

The strengths of the present study include taking into account diverse opinions and experiences expressed by the multidisciplinary panel, the large panel size, which may have improved transparency, our real-time documentation of the decision-making process, and independent data collection and review of field notes by three investigators to minimize bias and error. Recognized limitations include the possible influence of professional status and expertise of panel members on aspects of evidence discussed (e.g., dominance of discussions by “powerful” members may skew viewpoints [34,35]), and lack of explicit documentation of panelists’ potential financial and intellectual conflicts of interest [36].

## 5 Conclusions

The present study gives insight into the many factors that influence the decision-making process in multidisciplinary guideline development groups. The findings also suggest that the process of the “Child Health Evidence Week” combined with the GRADE grid may aid transparency in the deliberative process of multidisciplinary guideline development groups in low-income settings, and provide a mechanism for comprehensive assessment, documentation, and reporting of the many factors that influence the quality and applicability of evidence-based guideline recommendations. Further studies on systematic methods to improve transparency and the rigor of how recommendations are derived from multiple evidence sources during guideline development are needed. In addition, a prospective study that assesses the impact of these methods for guideline development on patient’s outcomes is key.

## Figures and Tables

**Table 1:** Profile of guideline development panel members

Characteristic	Frequency	%
Sex		
Male	34	49
Female	36	51

Age (yr)		
21–30	8	11
31–40	35	50
41–50	14	20
51–60	10	14
Above 60	3	4

Profession		
Pediatrician	32	46
Medical officer	11	16
Nursing officer	7	10
Research (research supervision) role	5	7
Trainer of health care workers	5	7
National or provincial role for MoMSs/MoPHS	4	6
Clinical officer	3	4
Pharmacist	2	3
Academic administration	1	1

Number of years as a health care professional		
3–7	23	33
8–12	14	20
13–21	16	23
22–40	17	24

*Abbreviations:* MoMSs, Ministry of Medical Services; MoPHS, Ministry of Public Health and Sanitation.

**Table 2:** Clinical topics and themes

Clinical topic	Referral to various types of evidence		Assessment of evidence			Assessment of local applicability of evidence			
	Research evidence	Clinical experience	Quality of evidence	Nature of intervention	Interpretation of evidence	Feasibility	Local context	Balance of benefits/harms	Values and preferences
Hand washing	√	—	—	√	√	√	–	√	√
Sepsis—clinical signs	—	—	—	√	√	—	√	—	—
Sepsis—antibiotics	√	—	—	—	√	—	√	—	—
Pneumonia	√	—	√	√	√	√	√	—	√
Kangaroo care	√	√	—	√	√	√	√	√	—
Asthma	√	√	—	—	√	√	—	√	—
HIV—pneumonia	√	—	√	√	√	√	—	—	—
Feeding regimens	√	√	√	√	√	√	—	—	—
Malnutrition—fluids	√	√	√	√	√	—	—	—	—
Meningitis	√	√	√	√	√	√	√	—	—
Malnutrition—RTUFs	—	√	√	√	√	√	—	—	√

*Abbreviation:* RTUFs, ready-to-use therapeutic foods.

## Supplementary data

Appendices A–D
