# Supplementary material for: The “Child Health Evidence Week” and GRADE grid may aid transparency in the deliberative process of guideline development
Source: J Clin Epidemiol. 2012 Sep;65(9-10):962–9. doi: 10.1016/j.jclinepi.2012.03.004 (PMC3413881; doi:10.1016/j.jclinepi.2012.03.004)
Supplement: Appendices A–D [file mmc1.pdf]

Webappendix A. Clinical topics and proposed recommendations

Topic	Proposed recommendations†
**1. Handwashing**	• Handwashing before and after EACH contact with patients, and after contact with hospital surfaces / equipment, after removing gloves, before handling food / drugs should be made a standard of care for all health care workers in all hospitals<br>• Caretakers should wash their hands before and after EACH contact with a patient, after visits to the bathroom and after cleaning ward areas or spills in all inpatient areas of hospitals<br>• Alcohol handrubs should replace simple soap and water (or medicated soap and water) for hand hygiene in inpatient settings (note for visibly contaminated hands soap and water are to be used before applying alcohol to dry hands)<br>• Hand hygiene should be promoted using: education, posters, supervision and senior staff / management support at a minimum<br>• Medicated soaps should be recommended instead of simple soaps for staff handwashing in hospital areas
**2. Neonatal sepsis - Clinical signs**	• Children aged 0 to 59 days with one or more of the *11 signs or symptoms (Option '8+3')* should be recognised as being in a high-risk group for serious illness
**3. Neonatal sepsis - Antibiotics**	• Therapy with a 3rd generation Cephalosporin (Cefotaxime / Ceftriaxone) should replace Penicillin **plus** Gentamicin as empiric treatment of neonatal sepsis that is community acquired or birth canal-acquired
**4. Pneumonia**	• Benzyl penicillin / ampicillin should be replaced with oral amoxicillin for the treatment severe pneumonia<br>• Chloramphenicol should be abandoned as an alternative treatment for very severe pneumonia<br>• Co-trimoxazole should be replaced with amoxicillin for treatment of children with non-severe pneumonia<br>• Gentamicin should be added to currently recommended benzyl penicillin / ampicillin for treatment of severe pneumonia<br>• Currently recommended antibiotics should be replaced with ceftriaxone for treatment of very severe pneumonia
**5. Kangaroo care**	• Kangaroo mother care should be used in hospitals for care of Low birth weight infants AFTER STABILIZATION as an alternative to conventional methods of care<br>• Kangaroo mother care should be used in hospitals for care of Low birth weight infants BEFORE STABILIZATION as an alternative to conventional methods of care
**6. Asthma**	• Inhaled salbutamol should be used for the management of acute asthma in children <5 years<br>• Homemade bottle spacers should be used as the preferred alternative to commercial spacers in the delivery of inhaled

	bronchodilators<br>• Inhaled salbutamol is the recommended therapy for the management of acute asthma. *Aminophylline should not be used for the management of acute asthma*<br>• Guidelines should clearly state that inhaled therapy and steroids are 1st line. For **non-responsive patients** with life threatening asthma (after 3 nebulisers in 1 hour) second line therapy could include: iv aminophylline or iv salbutamol should be used only in level 4 or higher level setting ideally with an HDU (High Dependency Unit)/ICU (Intensive Care Unit) with monitoring
**7. HIV - Pneumonia**	• Ceftriaxone should be used as an alternative first line to ampicillin / penicillin plus gentamicin for treatment of HIV infected children with very severe pneumonia<br>• Use of empiric cotrimoxazole treatment for severe or very severe pneumonia in children should be restricted to children <1 year<br>• Adjuvant corticosteroid therapy NOT indicated in the treatment for HIV infected children with PCP (Pneumocystis carinii pneumonia)
**8. Neonatal feeding regimens**	• For babies weighing 1.0 to 1.5 kg starting feeds:<br>○ **Starting volume:** 5 mls / 3 hrly equal to 27 – 40 mls/kg/day<br>○ **Incremental feed volume:** Add 5 mls to previous day's 3 hourly feed volume, equal to increasing feed volume by 27 – 40 mls/kg/day, until max reached<br>• Feeding regimens should be left to the discretion of the clinician seeing the babies and volumes of enteral feeds in preterm / small for gestational infants should be decided daily and not follow a standard plan<br>• <u>All babies </u><1500g and all weighing >1500 g, with asphyxia or resp. distress - *Delay feeding for first 24 hours : Start feeding from birth all >1500g who are stable*
**9. Malnutrition - Fluids**	• Children with severe malnutrition and shock receive **modest resuscitation with isotonic fluids** - 10ml/kg over 15-30 minutes and repeated if shock persists and no evidence of fluid overload<br>• Children with severe malnutrition and shock receive **aggressive resuscitation with isotonic fluids** - 20ml/kg given rapidly and repeated if shock persists and no evidence of fluid overload. Maximum of 4 boluses with blood used if appropriate<br><br>Option 2 (moderate fluid resuscitation)<br>• Children with severe malnutrition and shock receive **aggressive resuscitation with isotonic fluids** - 20ml/kg given rapidly and repeated if shock persists and no evidence of fluid overload. Maximum of 4 boluses with blood used if appropriate

**10. Meningitis**	• Children aged ≥60 days presenting with fever and get a diagnosis of meningitis by LP (Lumbar Puncture) should be treated with Ceftriaxone
**11. Severe malnutrition (ready-to-use therapeutic foods)**	• Hospitalized children aged 6-59 months with severe malnutrition should be managed with RTUF (ready-to-use-therapeutic foods)

† Based on identified research evidence – the final recommendations were formulated following discussions on both research and contextual factors

Child and Newborn Health Group

Kenya Medical Research Institute wellcome trust

Evidence summary: Kangaroo mother care for low birth weight infants

Clinical need

Kangaroo mother care (KMC), defined as continuous skin to skin between a mother and her newborn allowing frequent and exclusive breastfeeding, has been proposed as an alternative to conventional care (incubators, cots, etc) for low birth weight (LBW, less than 2500 g) babies. The evidence for the effectiveness and safety of KMC versus conventional care in LBW infants is considered in this summary.

Clinical questions, Quality of evidence‡ and Key findings

- **Population:** Low birth weight infants, less than 2500 g
- **Comparisons:** Kangaroo mother care versus conventional care
- **Outcomes:** Neonatal mortality, morbidity, breastfeeding status, costs and length of hospital stay

**1. What is the evidence that KMC reduces the risk of mortality in LBW infants?**
Key findings<br>• **Low quality evidence** suggests that KMC does not reduce the risk of death in ***stabilized*** LBW infants<br>• **Low quality evidence** suggests that KMC may reduce the risk of death in LBW infants if initiated very early in life ***before stabilization***
**2. What is the evidence that KMC reduces morbidity in LBW infants?**
Key findings<br>• **Low quality evidence** suggests that KMC reduces the risk of morbidity (mild / illnesses, nosocomial infections) in LBW infants.
**3. What is the evidence that KMC improves breastfeeding outcomes in LBW infants?**
Key findings<br>• **Low quality evidence** suggests that KMC increases the likelihood of exclusive breastfeeding at discharge in LBW infants

- **Moderate quality evidence** suggests that KMC increases the likelihood of exclusive breastfeeding at 41 weeks corrected age in LBW infants
- **Very low quality evidence** suggests that KMC may improve the chances of exclusive breastfeeding of LBW infants at the age of six months post birth

4. What is the evidence that KMC reduces the length of hospital stay of LBW infants?

Key findings

- **Low quality evidence** suggests that LBW babies on KMC stay hospitalized for a shorter duration compared to those on conventional care

5. What is the evidence for the cost-benefit of KMC compared to standard neonatal care?

Key findings

- **Very low quality evidence** suggests that the cost of care for babies on KMC is lower than the costs of standard care

‡ Quality of evidence is categorized as 'high', 'moderate', 'low' or 'very low'.

- **HIGH**: Further research is very unlikely to change our confidence in the estimate of effect.
- **MODERATE:** Further research is likely to have an important impact on our confidence in the estimate of effect and may change the estimate.
- **LOW:** Further research is very likely to have an important impact on our confidence in the estimate of effect and is likely to change the estimate.
- **VERY LOW**: We are very uncertain about the estimate.

Quality of Evidence and Summary of Findings

Question 1: What is the evidence that KMC reduces the mortality risk in LBW infants?

Intervention: Kangaroo mother care
Comparison: Conventional care
Bibliography: Worku et al[3]; Charpak et al[4]; Sloan et al[5]; Cattaneo et al[5]; Suman et al[9]

Quality assessment							Summary of findings		Importance
							Effect size (95% CI)	Quality (GRADE)	
**No of studies**	**No of infants**	**Design**	**Limitations**	**Inconsistency**	**Indirectness**	**Imprecision**			
**Mortality before stabilization (follow-up 4 to 6 days)**									
1	123	randomised controlled trial	serious†	no serious inconsistency	no serious indirectness	serious‡	RR 0.57 (0.33 to 1.0)	⊕⊕⊖⊖ **LOW**	CRITICAL
**Mortality after stabilization (follow-up 1 to 7 weeks)**									
4	1512	randomised controlled trials	serious††	no serious inconsistency	no serious indirectness	no serious imprecision	RR 0.70 (0.41 to 1.21)	⊕⊕⊕⊖ **MODERATE**	CRITICAL

† - significant number of recruited infants not randomised, blinding of investigators / data collectors unclear; ‡ small sample size, wide 95% confidence interval; †† blinding of investigators / data collectors unclear, potential for reporting bias (selective reporting of outcomes)

Question 2: What is the evidence that KMC reduces morbidity in LBW infants?

Intervention: Kangaroo mother care
Comparison: Conventional care
Bibliography: Charpak et al[4] Sloan et al[5]; Cattaneo et al[6]; Charpak et al[7]

Quality assessment							Summary of findings		Importance
No of studies	No of infants	Design	Limitations	Inconsistency	Indirectness	Imprecision	Effect size (95% CI)	Quality (GRADE)	
**Morbidity (risk of severe illness at discharge)**									
1	285	randomised controlled trial	serious†	no serious inconsistency	no serious indirectness	serious‡	RR 0.51 (0.28 to 0.94)	⊕⊕⊖⊖ **LOW**	CRITICAL
**Morbidity (risk of infectious episodes at 40 to 41 weeks corrected age; follow-up 3 to 10 weeks)**									
1	746	randomised controlled trial	serious¶	no serious inconsistency	no serious indirectness	serious‡	RR 1.06 (0.72 to 1.54)	⊕⊕⊖⊖ **LOW**	CRITICAL
**Morbidity (risk of nosocomial infections at 40 to 41 weeks corrected age; follow-up 3 to 10 weeks)**									
1	285	randomised controlled trial	serious¶	no serious inconsistency	no serious indirectness	serious‡	RR 0.47 (0.30 to 0.73)	⊕⊕⊖⊖ **LOW**	CRITICAL
**Morbidity (risk of severe illness at 6 months (follow-up 0-6 months)**									

1	275	randomised controlled trial	serious¶	no serious inconsistency	no serious indirectness	serious‡	RR 0.90 (0.30 to 0.66)	⊕⊕⊖⊖ **LOW**	CRITICAL
**Morbidity (risk of severe illness at 1 year corrected age; follow-up 0 to 12 months)**									
1	285	randomised controlled trial	serious††	no serious inconsistency	no serious indirectness	serious‡	RR 0.95 (0.06 to 15.09)	⊕⊕⊖⊖ **LOW**	CRITICAL

† - blinding of intervention to both investigators and data collectors unclear; ‡ - few number of events; ¶ - unclear concealment of intervention allocation, unblinded outcome assessment; †† - significant loss to follow-up

Question 3: What is the evidence that KMC improves breastfeeding outcomes in LBW infants?

Intervention: Kangaroo mother care
Comparison: Conventional care
Bibliography: Cattaneo et al[6]; Hake-Brooks et al[11]; Rojas et al[12]; Boo et al[13]

**Quality assessment**							**Summary of findings**		**Importance**
**No of studies**	**No of infants**	**Design**	**Limitations**	**Inconsistency**	**Indirectness**	**Imprecision**	**Effect size (95% CI)**	**Quality (GRADE)**	
**Breastfeeding (exclusively at discharge; follow-up 0 to 30 days)**									
4	537	randomised controlled trial	serious†	no serious inconsistency	serious‡	no serious imprecision	RR 1.33 (1.17 to 1.5)	⊕⊕⊖⊖ **LOW**	IMPORTANT
**Breastfeeding (exclusively at 40 to 41 weeks corrected age; follow-up 0 to 10 weeks)**									
1	746	randomised controlled trial	serious†	no serious inconsistency	no serious indirectness	no serious imprecision	RR 1.02 (0.87 to 1.21)	⊕⊕⊕⊖ **MODERATE**	IMPORTANT
**Breastfeeding (exclusively at 6 months of age; follow-up mean 6 months)**									
1	66	randomised controlled trial	serious¶	no serious inconsistency	serious‡	serious††	RR 2.51 (0.11 to 59.53)	⊕⊖⊖⊖ **VERY LOW**	IMPORTANT
**Breastfeeding (follow-up 0 to 12 months)**									

2	759	randomised controlled trial	serious†	no serious inconsistency	serious‡	serious††	RR 0.92 (0.69 to 1.23)	⊕⊖⊖⊖ **VERY LOW**	IMPORTANT

† - unclear concealment of allocation of interventions / blinding of outcome assessment; ‡ - one of the included studies conducted in a high income setting with a lactation consultant; ¶ - unclear concealment of allocation of interventions; †† - small number of events (<300)

Question 4: What is the evidence that KMC reduces the length of hospital stay of LBW infants?

Intervention: Kangaroo mother care
Comparison: Conventional care
Bibliography: Kadam et al[8]; Gathwala et al[10]; Boo et al[13]

Quality assessment							Summary of findings		Importance
No of studies	No of infants	Design	Limitations	Inconsistency	Indirectness	Imprecision	Effect size (95% CI)	Quality (GRADE)	
**Length of hospital stay (follow-up 0 to 40 days)**									
1	126	randomised controlled trial	serious†	no serious inconsistency	no serious indirectness	serious‡	13.5 days (KMC) versus 22.5 days (CMC)	⊕⊕⊖⊖ **LOW**	IMPORTANT
**Length of hospital stay (follow-up 0 to 2 months)**									
2	199	randomised controlled trial	serious†	serious¶	no serious indirectness	serious‡	KMC = shorter duration of hospital stay††	⊕⊖⊖⊖ **VERY LOW**	IMPORTANT

† - unclear concealment of allocation of interventions / blinding of outcome assessment /selective reporting of outcomes; ‡ - small number of enrolled participants; CMC - conventional method of care ¶ - age at randomisation into KMC group inconsistent across included studies; †† - Gathwala et al; (KMC, 3.56 days versus CMC, 6.8 days), Kadam et al (KMC, 8.5 days versus CMC, 9.3 days)

Question 4: What is the evidence that KMC reduces the length of hospital stay of LBW infants?

Intervention: Kangaroo mother care
Comparison: Conventional care
Bibliography: Sloan et al[5]; Cattaneo et al[6]

**Quality assessment**							**Summary of findings**		**Importance**
**No of studies**	**No of infants**	**Design**	**Limitations**	**Inconsistency**	**Indirectness**	**Imprecision**	**Effect size (95% CI)**	**Quality (GRADE)**	
**Cost of care (follow-up 0 to 6 months)**									
2	560	observational studies	very serious†	no serious inconsistency	serious‡	no serious imprecision	KMC = Lower costs of care¶	⊕⊖⊖⊖ **VERY LOW**	IMPORTANT

† - potential for selection / investigator bias in recruitment of participants / measurement of outcomes; ‡ - items costed unclear; ¶ - Sloan et al (KMC, US $101 versus CMC, US $130), Cattaneo et al (KMC, US $7,501 versus CMC, US $9,876)

Characteristics of the evidence

This evidence summary is based on a comprehensive search and critical appraisal (for methodological rigor and clinical practice applicability) of best currently available literature. The evidence in this summary comes from:

- One Cochrane review of randomised controlled trials (RCTs) (N=1,362 infants, 3 studies)[1]
- One overview of 2 systematic reviews and 7 RCTs[2]
- Ten RCTs (N=2,086 infants)[3-12]

References

1. Conde-Agudelo A, Belizán JM. *Kangaroo Mother Care to reduce morbidity and mortality in low birth weight infants.* Cochrane Database Syst Rev. 2003; (2).
2. Blackwell K, Adriano C. *What is the evidence of Kangaroo mother care of the* very low *birth weight infants*. International Child Health Review Collaboration (ICHRC). Available at: www.ichrc.org/pdf/kangaroo.pdf, Accessed 17th May 2010.
3. Worku, B, Kassie A. *Kangaroo Mother Care: A randomized controlled trial on effectiveness of early kangaroo mother care for the low birth weight infants in Addis Ababa, Ethiopia.* J Trop Ped, 2005: 51(2); 93-7
4. Charpak N, Ruiz-Pelaez JG, *et al*. *A randomized, controlled trial of kangaroo mother care: results of follow-up at 1 year of corrected age.* Pediatrics, 2001:108(5); 1072-1079.
5. Sloan NL, Camacho LW, Rochas EP*, et al*. *Kangaroo mother method: randomised controlled trial of an alternative method of care for stabilized low-birthweight infants.* Maternidad Isidro Ayora Study Team. Lancet, 1994: 344(8925); 782-785.
6. Cattaneo A, Davanzo R, Worku B, *et al*. *Kangaroo mother care for low birth weight infants: a randomised controlled trial in different settings.* Acta Paed, 1998: 87; 976-985.
7. Charpak N, Ruiz-Palaez JG, *et al. Kangaroo mother versus traditional care for newborn infants < 2000 grams: a randomised controlled trial.* Pediatrics, 1997:100(4):682-688.
8. Kadam S, Binoy S, Kanbur W, *et al*. *Feasibility of kangaroo mother care in Mumbai.* Indian J Ped, 2005:72; 35-38.
9. Suman RP, Udani R, Nanavati R. *Kangaroo mother care for low birth weight infants: a randomized controlled trial*. Indian Pediatr, 2008: 45; 17–23.
10. Gathwala G, Singh B, Balhara B. *KMC facilitates mother baby attachment in low birth weight* infants. Indian J Ped, 2008: 75; 43-47.
11. Hake-Brooks SJ, Anderson S. *Kangaroo mother care and breast-feeding of mother-preterm infant dyads 0-18 months: A randomized, controlled trial.* Neonatal Network, 2008: 27; 151-159.
12. Rojas MA, Kaplan M, Quevedo M, *et al*. *Somatic growth of preterm infants during skin-to-skin care versus traditional holding: A randomized, controlled trial.* Developmental and Behavioral Pediatrics, 2003: 24; 163–168.

Figure(s)

Appendix C. Sample GRADE grid used to record participants votes during the development of pneumonia recommendations

**Current recommendation**	At present, benzyl penicillin or parenteral ampicillin is recommended for treatment of WHO-defined severe pneumonia in children aged 2 to 59 months
**Proposed recommendation**	Benzyl penicillin / ampicillin should be replaced with oral amoxicillin for the treatment severe pneumonia
**Quality of research evidence**	**Moderate to high quality evidence** from three randomized controlled trials suggests similar clinical outcomes in children with severe pneumonia treated inpatient with amoxicillin and benzyl penicillin/ampicillin
**ARE THERE FACTORS OTHER THAN THE EVIDENCE TO CONSIDER?**	
Benefits or desired effects – to patients, to staff?	Safety of oral over injectable treatments, convenient dosing schedule (twice daily versus four times a day)
Risks or undesired effects – to patients, to healthcare staff?	Amoxicillin may not be as effective as benzyl penicillin in African children
Costs	Potential reduction in cost of resources required for injectable treatment including the option of out-patient management
Feasibility	Amoxicillin is widely available
Values and preferences	Painless oral route for amoxicillin preferable to injections required for benzyl penicillin/ampicillin

**SCORE†**				
Strongly recommend against proposal	Weakly recommend against proposal	Neither recommend for or against proposal	Weakly recommend proposal	Strongly recommend proposal
-2	-1	0	1	2
☐	☐	☐	☐	☐

† Participants recorded their judgments on a scale of -2 to +2 following discussions on the provided research evidence and relevant contextual factors

Appendix D. Voting results for pneumonia recommendation

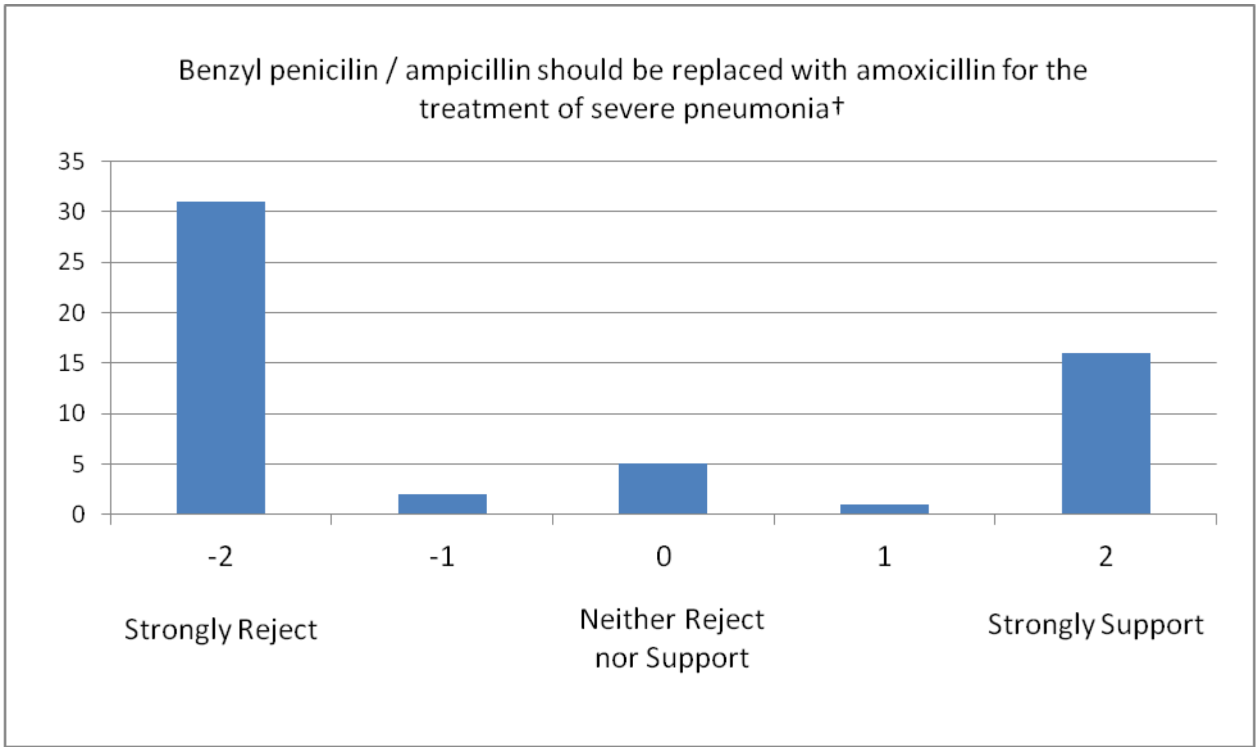

†Evidence quality: **moderate quality evidence** in support of proposed recommendation. Participants voted against the proposed recommendation probably due to indirectness of evidence [14]
